# Data on fluoride contents in groundwater of Bushehr province, Iran

**DOI:** 10.1016/j.dib.2018.02.016

**Published:** 2018-02-13

**Authors:** Sina Dobaradaran, Maryam Khorsand, Abdolreza Hayati, Roya Moradzadeh, Mohammad Pouryousefi, Mostafa Ahmadi

**Affiliations:** a The Persian Gulf Marine Biotechnology Research Center, The Persian Gulf Biomedical Sciences Research Institute, Bushehr University of Medical Sciences, Bushehr, Iran; b Systems Environmental Health, Oil, Gas and Energy Research Center, The Persian Gulf Biomedical Sciences Research Institute, Bushehr University of Medical Sciences, Bushehr, Iran; c Department of Environmental Health Engineering, Faculty of Health, Bushehr University of Medical Sciences, Bushehr, Iran; d Department of Environmental Engineering Bushehr branch, Islamic Azad University, Bushehr, Iran; e Urban Water and Wastewater Company, Bushehr Province, Iran

**Keywords:** Bushehr, Drinking water, Fluoride, Groundwater, Spectrometer

## Abstract

In this article, we measured the levels of fluoride in groundwater. The samples were taken from groundwater in Bushehr's province, Iran. After the collection of samples, the concentration levels of fluoride were determined by the standard SPADNS method using spectrometer. The mean concentration levels of fluoride in water of all stations were higher than the WHO drinking water guideline. Microsoft Office Excel 2016 was used for calculation of mean values. The mean concentration level of fluoride instatement were in the range of 1.52 to 3.64 mg l^-1^.

**Specifications Table**

**Table:** 

*Subject area*	*Environment*
*More specific subject area*	*Fluoride*
*Type of data*	*Table, figure*
*How data was acquired*	*Spectrophotometer*
*Data format*	*Raw, analyzed*
*Experimental factors*	*All water samples in polyethylene were stored in a dark place at* room *temperature until the fluoride analysis.*
*Experimental features*	*Determine the concentration levels of fluoride*
*Data source location*	*Bushehr province, Iran*
*Data accessibility*	*Data is available within this article*.

**Value of the data**

• The maximum and minimum daily intake of fluoride based on 2 l daily water on consumption reached 3.04 and 7.88 mg day^-1^ respectively.
• The data presented here showed the Removal of high concentration of fluoride (F) from drinking water is necessary in this region and measures should be taken to supply water after removal of fluoride for the betterment of the livelihood in the area.
• Data shown here may serve as benchmarks for other groups working or studying in the field of pollution control, aquatic ecosystem.

## 1 Data

In the data, as shown in Table 1, the mean±SD concentration levels of fluoride in groundwater samples in all station samples were 2.08 ± 0.7 mg l^-1^. The lowest and highest F concentration levels were 1.52 mg l^-1^ and 3.94 mg l^-1^ in samples S_19_ (Kangan) and S_4_ (Tange eram) respectively. As shown in Table 2, the concentration levels of fluoride in water of all stations were higher than the WHO and USA, UK, Canada drinking water guidelines for fluoride. As seen Table 1, it shows that the mean value daily intakes of fluoride based on 2 l daily drinking water consumption reach 0.36 mg day^-1^with a range of 0–0.96 mg day^-1^.

**Table 1:** Concentrations of fluoride (mg l^-1^) in groundwater samples of measured stations (maximum values are expressed as bold italics; minimum values as bold underlined).

Station	**Location**	**Fluoride concentration (mg l**^**-1**^**)**	**Daily intake (mg day**^**-1**^**)**	**X**	**Y**
S_1_	Anarestan	2.1	4.2	605,849	3,101,575
S_2_	Bushkan	3.18	6.36	569,098	3,189,677
S_3_	Tange eram	3.66	7.32	550,690	3,225,304
S_4_	Tange eram	*3.94*	*7.88*	550,744	3,252,570
S_5_	Khormooj	1.83	3.66	537,788	3,168,812
S_6_	Khormooj	1.76	3.52	538,788	3,169,065
S_7_	Khormooj	2.44	4.88	538,661	3,169,531
S_8_	Khormooj	2.44	4.88	540,084	3,173,759
S_9_	Khormooj	1.71	3.42	538,650	3,169,533
S_10_	Dayyer	1.54	3.08	595,733	3,088,740
S_11_	Shonbe	1.84	3.68	575,367	3,141,132
S_12_	Shonbe	1.72	3.44	575,297	3,141,223
S_13_	Kaki	1.63	3.26	552,561	3,136,027
S_14_	Kaki	1.61	3.22	552,876	3,136,136
S_15_	Kaki	1.62	3.24	552,719	3,135,654
S_16_	Kaki	1.63	3.26	552,845	3,135,284
S_17_	Kalame	1.72	3.44	546,346	3,197,877
S_18_	Kangan	1.53	3.06	601,058	3,084,484
S_19_	Kangan	**1.52**	**3.04**	604,915	3,080,492
S_20_	Kangan	1.62	3.24	602,845	3,087,254
S_21_	Kangan	2.74	5.48	604,010	3,081,587
Mean ± SD		**2.08±0.7**	**4.16±1.4**		
Median		**1.72**	**3.44**		

^*^Based on 2 l daily drinking water consumption and concentration levels of fluoride in drinking waters.

**Table 2:** Different drinking water quality guidelines for fluoride.

**Drinking water quality guidelines**	**Fluoride (mg l^-1^)**	**Reference**
**WHO**	0.5–1.5	[1]
**USA**	0.7–1.2	[2]
**Canada**	0.8–1.0	[3]
**UK**	0.3–0.7	[3]

## 2 Experimental design, materials and methods

### 2.1 Study area description

**Fig. 1:**
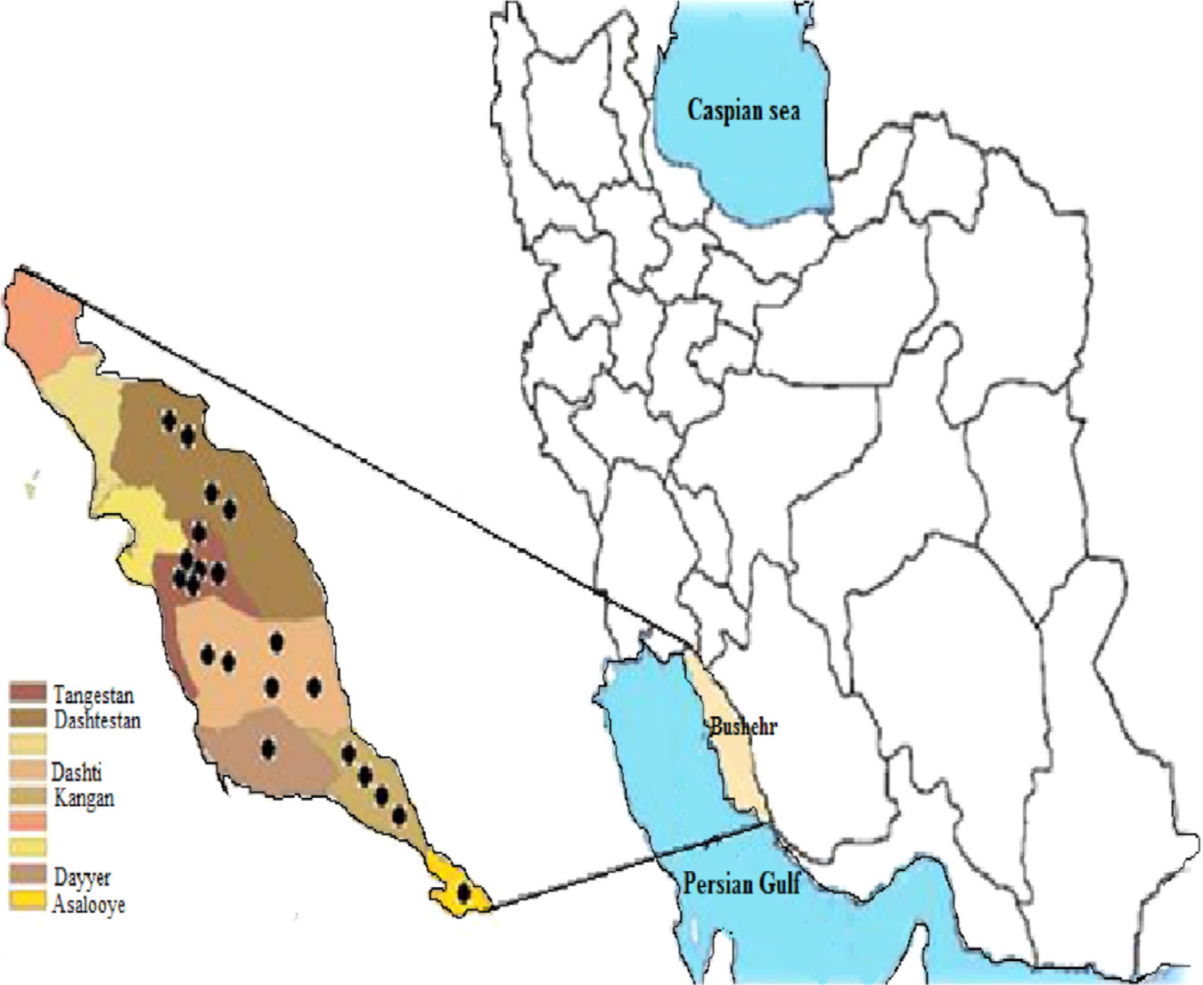
Locations of groundwater sample stations.

Nine town in Bushehr province, Iran were selected as sampling points including Anarestan, Bushkan, Tange eram, Khormouj, Dayyer, Shonbe, Kaki, Kalame and Kangan (Fig. 1).

### 2.2 Sample collection and analytical procedures

Water samples were collected by using 200 mL polyethylene bottles that were washed three times with deionized water; prior to collecting each sample, and then bottles were labeled with the sample number and location for identification. All samples were stored in a dark place at room temperature until analysis. After that, for the fluoride analyses, the SPADNS colorimetric method was used with a spectrometer [4], [5], [6], [7], [8], [9], [10], [11], [12]. Daily fluoride intakes were calculated based on 2 l daily drinking water consumption and concentration levels of fluoride in waters. Microsoft Office Excel 2016 was used for calculation of mean values.
