# Supplementary material for: Data on fluoride contents in groundwater of Bushehr province, Iran
Source: Data Brief. 2018 Feb 13;17:1158–62. doi: 10.1016/j.dib.2018.02.016 (PMC5988413; doi:10.1016/j.dib.2018.02.016)
Supplement: Supplementary file 1 — Supplementary material [file mmc1.docx]

**Dear Editor of Data in Brief**

The research reported in this manuscript has been funded through the Bushehr Urban Water and Wastewater Company. This manuscript has not been previously published and is not under consideration in the same or substantially similar form in any other peer-reviewed media. All authors listed have contributed sufficiently to the project to be included as authors. All authors have read the manuscript and have agreed to submit it in its current form for consideration for publication in the Journal.

**The authors declare that they have no conflict of interest, financial or other.**

Sincerely Yours,

Maryam Khorsand

Water and Wastewater Company, Raeis Ali Delvari Street, Bushehr, Iran,

Tel. /fax: +98 772527004, Iran.

E-mail: Khorsand.m65@gmail.com
